# Association between fluoroquinolones and retinal detachment: insights from a large German health claims-based cohort study

**DOI:** 10.1186/s12886-025-04284-5

**Published:** 2025-08-07

**Authors:** Julia Wicherski, Jonas Peltner, Cornelia Becker, Katrin Schüssel, Gabriela Brückner, Andreas Schlotmann, Helmut Schröder, Winfried V. Kern, Britta Haenisch

**Affiliations:** 1 Present Address: Research Division, Pharmacoepidemiology, Federal Institute for Drugs and Medical Devices (BfArM), 53175 Bonn, Germany; 2 German Center for Neurodegenerative Diseases (DZNE), Pharmacoepidemiology in Neurodegenerative Disorders, 53127 Bonn, Germany; 3 Research Institute of AOK (WIdO), Berlin, 10178 Germany; 4 Division of Infectious Diseases, Department of Medicine II, Faculty of Medicine and Medical Centre, University of Freiburg, 79106 Freiburg, Germany; 5 Center for Translational Medicine, University of Bonn, 53113 Bonn, Germany

**Keywords:** Active comparator new user design, Administrative cohort, Real-world data, Fluoroquinolones, Adverse drug reactions

## Abstract

**Purpose:**

Real-world evidence on fluoroquinolone-associated retinal detachment is contradictory. Therefore, we aim to examine the association between newly prescribed fluoroquinolones and the occurrence of retinal detachment with recent data from a large central European country.

**Methods:**

A cohort study with an active comparator new user design was conducted. Dispensings of fluoroquinolone episodes were compared to a group of reference antibiotic episodes (amoxicillin, amoxicillin clavulanic acid, azithromycin, cefuroxime, cephalexin, clindamycin, sulfamethoxazole-trimethoprim, and doxycycline). Data from one of the largest statutory health insurances in Germany, the AOK, were used to follow up individuals with new antibiotic dispensing during the years 2014–2018 for the occurrence of retinal detachment. Piece-wise exponential additive mixed models adjusted for person-time, age, gender, comorbidities, year, and quarter at index were applied to estimate adjusted hazard ratios (aHR) with corresponding 95% confidence intervals (95% CI).

**Results:**

In total, 15,232,585 antibiotic episodes were included in the cohort of which 0.05% episodes had an incident retinal detachment. The covariate-adjusted hazard ratio for fluoroquinolone episodes was 1.01 [0.95;1.08]. Likewise, in the propensity score-matched cohort the covariate-adjusted hazard ratio was 0.99 [0.92;1.07]. Moreover, there was little evidence for differences in age and gender subgroups, by follow-up time, selection of active comparator agent, dosage category, or censoring approach.

**Conclusions:**

This large German cohort study found no meaningful real-world evidence for the association between fluoroquinolones and retinal detachment compared to a group of active comparator antibiotics.

**Supplementary Information:**

The online version contains supplementary material available at 10.1186/s12886-025-04284-5.

## Introduction

Despite the evidence on serious adverse drug reactions and despite being declared a reserve antibiotic agent, fluoroquinolones (FQ) continue to be frequently used [1]. Due to their high efficacy, FQ are relevant antibiotics with a broad antimicrobial spectrum. Therefore, it is important to have comprehensive evidence on the safety of FQ treatment. In consequence to the risk assessment report of the European Medicines Agency (EMA), authorisation of FQs was restricted and changed in 2019 based on the high risk of FQ for several serious adverse events, such as aortic aneurysm or tendon ruptures [2], but the current real-world evidence on FQ-associated retinal detachment is inconsistent so far [3, 4].

The pathological mechanism of FQ’s association with retinal detachment is suspected to be collagen-associated just as aortic aneurysms and tendon ruptures are collagen-associated. FQs have the potential to damage collagen structures by activating the matrix metalloproteinase [5–7]. Collagen structures were found in the vitreous body and retina [8, 9]. Another pathophysiological mechanism discussed in the literature is the strong binding of levofloxacin to melanin. Melanin-containing ocular tissue could be influenced in its functionality by FQ exposure and subsequently cause retinal detachment [10]. Irrespective of the potential mechanisms, there is a gap of knowledge regarding differences in susceptibility for retinal detachment by age and gender and relative effects of FQ versus different comparator antibiotics. Information based on European routine data is also scarce.

Therefore, we designed a cohort study with recent health insurance data from Germany to contribute to real-world evidence of FQ safety in scope of retinal detachment.

## Methods

### Source of data

The “AOK–Die Gesundheitskasse” (AOK) covers approximately 28 million individuals in Germany [11]. AOK is therefore one of the German statutory health insurance providers with the largest number of members and data routinely collected by AOK are well-suited for analyses on a population-based level. The longitudinal billing-relevant data used for this cohort study cover information on date of birth and self-reported gender as well as medical diagnoses (classified by the German version of the International Statistical Classification of Diseases and Related Health Problems, 10th revision, ICD-10-GM) on a quarterly level for outpatient and on a weekly level for inpatient ICD-10-GM codes. Inpatient stays were also captured as well as OPS (the German adaptation of the International Classification of Procedures in Medicine) codes provided for procedures during hospitalisation) [12]. Moreover, the date on which prescribed drugs were dispensed, the anatomic-therapeutic-chemical (ATC) code (German adaptation of the World Health Organization’s ATC classification [12]), and the defined daily doses (DDD) were provided.

### Cohort

**Fig. 1:**
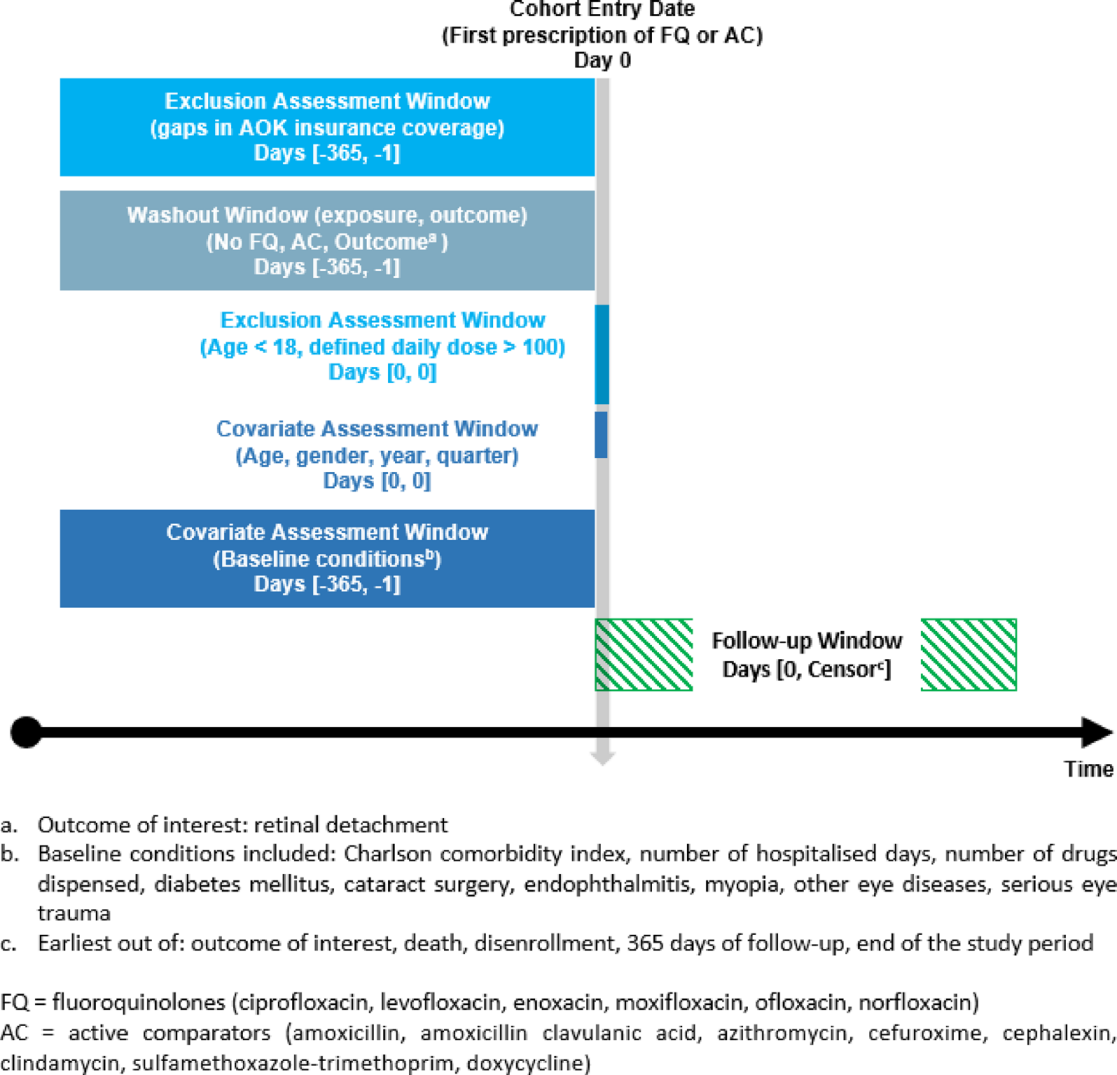
Study design diagram (template by Schneeweiss et al. [13])

This cohort is based on the period from 1 st January 2013 to 31 st December 2019. Index antibiotic episodes were identified as an initially (newly) dispensed prescription of systemic FQ or an active comparator drug (AC) after a 365-day wash-out period to ensure no previous antibiotic dispensings (Fig. 1). Topical FQ preparations were not considered as index antibiotic episodes. The date of the index antibiotic episode was established as the cohort entry date (CED). To only capture incident diagnoses of retinal detachment and avoid prevalent cases, the 365-day wash-out period was also applied to exclude patients with former diagnoses for retinal detachment. To be included, individuals required gapless AOK coverage for a 365-day baseline period. Only adults (i.e. ≥ 18 years at CED) were included. Moreover, antibiotic episodes with implausibly high doses (i.e., DDD > 100) were excluded. Due to the quarterly-based outpatient diagnoses, individuals with retinal detachment diagnoses in the index (CED) quarter were also excluded.

If all selection criteria were met, an individual was allowed to be included in the cohort at multiple time points during the study period (i.e. with multiple antibiotic episodes); the individual follow-up period was a risk window up to 365 days per episode.

The new occurrence of retinal detachment was detected by ICD-10-GM codes and corresponding OPS codes for surgical procedures (see eTable 1) from quarterly-based outpatient diagnoses with a “confirmed” label or calendar week-based inpatient discharge diagnoses. During the 365-day baseline window, the Charlson comorbidity index (CCI), number of drugs dispensed, and inpatient stays were assessed as general measures of comorbidity and frailty of individuals included. Moreover, specific comorbidities related to retinal detachment (eTable 1) were assessed during the baseline period.

### Statistical analysis

Standardised mean differences were used to quantify balance of all baseline characteristics distribution between the exposure and reference group. German Census 2011 [14] was defined as the standard population to estimate age- and gender-standardised incidence rates and their corresponding 95% confidence intervals (95% CI) by direct standardisation via Poisson approximation [15].

A 365-day risk window is a long period in which non-proportionalities between groups could easily occur. Therefore, the Piece-wise exponential additive mixed models (PAMM) [16] instead of a Cox regression model was applied. PAMM were applied to estimate covariate-adjusted hazard ratios (aHR) with baseline hazards modelled as a smooth, non-linear function. Age in years and individual person-time of index episodes were included in the model as smooth, non-linear time-constant effects whereas retinal detachment-related comorbidities, CCI, number of drugs dispensed, hospitalised days as well as CED year and quarter were modelled as linear time-constant effects.

Furthermore, several sensitivity and subgroup analyses were conducted. Nearest neighbour propensity score matching with a matching ratio of 1:1 and calliper of 0.1 was used to improve balance in baseline characteristics between FQ and AC episodes. Supplemented eTable 1 lists all variables included in the matching process. In other sensitivity analyses, exposure misclassification was examined in several ways. Firstly, by censoring follow-up time at the time point of a new antibiotic dispensing (“per-protocol” censoring). Secondly, individuals with baseline hospitalisation were excluded in order to exclude episodes where antibiotic exposure may have occurred during an inpatient stay, as information on inpatient antibiotic exposure is not available in German routine health care data. For the same reason, individuals were disaggregated by the number of hospitalised days during follow-up time in a third sensitivity analysis. As the pathophysiological mechanism could mainly explain rhegmatogenous causes of retinal detachment, we performed a sensitivity analysis disaggregated for the ICD-10-GM codes of retinal detachment. Moreover, gender and age subgroups were separately analysed. Likewise, dose categories were stratified on a drug-specific level based on the DDDs of the antibiotic prescriptions into low medium and high dose categories. Furthermore, we conducted separate analyses based on inpatient outcome diagnoses only since outpatient diagnoses were only available on a quarterly information level. By this analysis, we strove to study the association between FQ exposure and retinal detachment with a higher temporal resolution (calendar week-based) to analyse shortened risk windows of ≤ 30, ≤ 60, and ≤ 92 days, respectively. Lastly, pairwise PAMMs for FQ versus single comparisons of active comparator drugs were applied.

All analyses were carried out between March 2023 and December 2024. Propensity score matching was conducted in SAS, version 9.4. All other statistical analyses were conducted in R, version 4.1.0.

## Results

The cohort comprised 15,232,585 antibiotic index episodes, which fulfilled the outcome-specific selection criteria. Among these, 2,925,881 (19.21%) were FQ episodes and 12,306,704 (80.79%) were active comparator episodes. All baseline characteristics of the cohort are displayed in Table 1.

**Table 1:** Study population characteristics

	FQ	AC	Standardised difference
	(*n* = 2,925,881)	(*n* = 12,306,704)	
Age (mean (SD))	59.45 (19.47)	51.21 (19.53)	0.423
Male gender (%)	1,238,182 (42.32)	5,667,479 (46.05)	0.075
CCI (%)			0.267
0	1,471,844 (50.30)	7,618,869 (61.91)	
1–2	832,472 (28.45)	3,093,805 (25.14)	
3–4	380,748 (13.01)	1,053,137 (8.56)	
5+	240,817 (8.23)	540,893 (4.40)	
Drugs dispensed (%)			0.331
0	357,414 (12.22)	2,368,529 (19.25)	
1–3	498,276 (17.03)	2,864,477 (23.28)	
4–10	641,087 (21.91)	2,840,148 (23.08)	
11–20	539,983 (18.46)	1,838,440 (14.94)	
21+	889,121 (30.39)	2,395,110 (19.46)	
Hospitalised days (%)			0.215
0	2,142,258 (73.22)	9,994,576 (81.21)	
1–7	376,439 (12.87)	1,353,386 (11.00)	
8+	407,184 (13.92)	958,742 (7.79)	
CED year (%)			0.141
2014	656,302 (22.43)	2,352,127 (19.11)	
2015	640,315 (21.88)	2,417,278 (19.64)	
2016	604,220 (20.65)	2,459,358 (19.98)	
2017	540,741 (18.48)	2,503,015 (20.34)	
2018	484,303 (16.55)	2,574,926 (20.92)	
CED quarter (%)			0.077
Q1 (Jan.-Mar.)	899,151 (30.73)	4,070,976 (33.08)	
Q2 (Apr.-Jun.)	650,033 (22.22)	2,658,405 (21.60)	
Q3 (Jul.-Sep.)	656,153 (22.43)	2,421,069 (19.67)	
Q4 (Oct.-Dec.)	720,544 (24.63)	3,156,254 (25.65)	
Diabetes mellitus (%)	688,165 (23.52)	1,970,258 (16.01)	0.189
Cataract surgery (%)	74,530 (2.55)	200,633 (1.63)	0.064
Endophthalmitis (%)	851 (0.03)	2,875 (0.02)	0.004
Myopia (%)	6,652 (0.23)	22,668 (0.18)	0.010
Other eye diseases (%)	389,492 (13.31)	1,101,922 (8.95)	0.139
Serious eye trauma (%)	8,200 (0.28)	32,913 (0.27)	0.002

Standard deviation (SD) | fluoroquinolone (FQ) | active comparator (AC) | Charlson Comorbidity Index (CCI) | cohort entry date (CED) | Quarter 1–4 (Q1-4): January-March, April-June, July-September, October-December

There was an imbalance in the mean age of individuals with FQ episodes compared to individuals with AC episodes (standardised difference 0.423). The FQ group was on average 59 years old compared to 51 years in the AC group. Self-reported gender was comparable between groups (with 42% males in FQ episodes compared to 46% males in AC episodes). Moreover, individuals with FQ episodes tend to have a higher baseline frailty, as indicated by imbalances in CCI, number of drugs dispensed and hospitalised days during baseline. Accordingly, FQ episodes had a higher percentage of CCI-relevant comorbidities. Regarding CED year and quarter, there were no or only small differences between FQ episodes and AC episodes. However, there is a decreasing trend in FQ utilization over time: while episodes from 2014 contributed to 22% of all FQ episodes, this proportion declined to less than 17% in 2018. Moreover, during winter months (e.g. Q1, January – March, and Q4, October – December), more index antibiotic dispensings were observed. Regarding outcome-specific comorbidities, there were only small differences between FQ episodes and AC episodes. Patients with FQ episodes tended to be more often diagnosed with diabetes mellitus and other eye diseases.

### Primary outcome

During the 365-day risk window, 0.05% of all index episodes had an incident retinal detachment diagnosis. Among these, 1,624 diagnoses occurred during FQ episodes compared to 5,645 diagnoses during AC episodes. The cumulative incidence of retinal detachment was 5.16 diagnoses per 10,000 individuals with FQ compared to 4.23 diagnoses for AC. After age- and gender-standardisation, there was a small difference in the incidence between both groups (4.18 compared to 4.12 diagnoses per 10,000 episodes). The covariate-adjusted hazard ratio within the 365-day risk window was 1.01 [95%CI 0.95;1.08] in the unmatched cohort. Table 2 shows the result of the covariate-adjusted PAMM regression model of the main model.

**Table 2:** PAMM regression main analysis

	aHR	[95% CI]
FQ-episode (ref. AC)	1.01	[0.95;1.08]
Males (ref. females)	1.39	[1.32;1.46]
CCI (ref. 0)		
1–2	0.99	[0.93;1.05]
3–4	0.97	[0.89;1.05]
5+	0.89	[0.79;1.00]
Drugs dispensed (ref. 0)		
1–3	1.23	[1.10;1.39]
4–10	1.29	[1.15;1.44]
11–20	1.26	[1.12;1.42]
21+	1.19	[1.06;1.35]
Hospitalised days (ref. 0)		
1–7	1.11	[1.03;1.20]
8+	1.15	[1.05;1.25]
CED year (ref. 2014)		
2015	1.05	[0.97;1.14]
2016	1.01	[0.93;1.10]
2017	1.10	[1.02;1.19]
2018	1.09	[1.00;1.18]
CED quarter (ref. Q1 (Jan.-Mar.)		
Q2 (Apr.-Jun.)	1.00	[0.93;1.07]
Q3 (Jul.-Sep.)	0.98	[0.91;1.06]
Q4 (Oct.-Dec.)	1.03	[0.96;1.10]
Diabetes mellitus	0.93	[0.87;1.00]
Cataract surgery	2.50	[2.27;2.75]
Endophthalmitis	10.46	[7.50;14.59]
Myopia	3.05	[2.43;3.84]
Other eye diseases	2.75	[2.58;2.93]
Serious eye trauma	2.71	[2.11;3.50]

	edf	p-value
Approximate significance of smooth terms		
Follow-up	2.868	< 0.001
Age in years	5.728	< 0.001

Propensity score (PS) | adjusted hazard ratio (aHR) with corresponding 95% confidence interval [95%CI] | fluoroquinolone (FQ) | active comparator (AC) | Charlson Comorbidity Index (CCI) | cohort entry date (CED) | Quarter 1–4 (Q1-4): January-March, April-June, July-September, October-December | effective degrees of freedom (edf)

Males had a higher relative risk of experiencing retinal detachment than females. Individual person-time and age in years were relevant smoothing terms of the regression models (p-value < 0.001 displayed at the bottom of Table 2). Relative risk of retinal detachment increased with the number of drugs dispensed but for CCI the results were inconclusive. Whereas diabetes mellitus was associated with a slightly decreased relative risk, all other comorbidities were associated with a highly increased relative risk of retinal detachment, especially endophthalmitis (Table 2).

After the matching process, all baseline variables were well balanced between FQ episodes and AC episodes with each 2,925,881 episodes included in the propensity score-matched analysis (supplemented eTable 2). No differences were observed in comparison to the propensity score-matched cohort (supplemented eTable 3). Supplemented eTable 4 lists the effect estimates of FQ episodes in all other subgroup and sensitivity analyses conducted. Again, hazard ratios were close to 1 and the corresponding 95% CIs included 1 in almost all analyses. There is a nominally decreased effect estimate for ≤ 39 years old females with FQ episode relative to AC, but the 95% CI included an aHR of 1 (aHR = 0.57 [0.33;1.00]). Likewise, there is no difference between FQ episodes compared to single active comparators, except for one decreased relative risk estimate for FQ episodes compared to clindamycin based on inpatient diagnoses of retinal detachment during the 92 days risk window (aHR = 0.78 [0.63;0.97]) as it is shown in supplemented eTable 5.

## Discussion

In this cohort study with an active comparator new-user design based on German health insurance claims, the absolute difference in the risk of retinal detachment after FQ dispensing compared to several reference antibiotics was small and there was no evidence for an increased relative risk of retinal detachment overall. Except for two single comparisons, one for young females with FQ episodes relative to all AC, and one for the comparison of FQ to clindamycin based on inpatient diagnoses with 92-day risk window, no meaningful differences between age and gender or single active comparators could be found. Furthermore, no dose-response relationship was observed.

Previous observational studies regarding FQ-associated retinal detachment reported conflicting findings but overall, the more recent self-controlled case series and cohort studies showed no evidence for an association between FQ exposure and retinal detachment occurrence. However, there are three studies reporting an increased relative risk of FQ for retinal detachment: The Canadian nested case-control study conducted by Etminan et al. [17] observed 4,384 retinal repair cases and reported an adjusted rate ratio of 4.50 [3.56;5.70] for current FQ use defined as prescriptions overlapping the index date (i.e. retinal detachment and repair). For recent (1–7 days) and past use (8-365 days), this study also found no differences [17]. The cohort of ≥ 65-year-old Canadians by Daneman et al. [18] estimated an increased adjusted hazard ratio of 1.47 [1.08;2.00] for FQ use compared to no use, but for the negative tracer exposure to amoxicillin the same aHR was estimated, resulting in no difference between FQ and amoxicillin, which is in line with our results showing no elevated relative risks of FQ compared to amoxicillin. A cohort study from Taiwan comparing FQ to amoxicillin reported an increased relative risk, too. During their 90-days risk window, 142 individuals experienced rhegmatogenous retinal detachment (aHR = 2.07 [1.45;2.96]) in their propensity score-matched cohort [19].

In comparison to this study, our cohort study provided evidence based on a larger number of cases of retinal detachment with the same absolute risk of FQ exposure but the risk of our reference antibiotics was equal to FQ exposure. The US data-based study by Fife et al. [20] replicated Etminan’s study twice and estimated adjusted odds ratios for any FQ exposure of 1.17 [1.09;1.26] and 1.22 [1.09;1.38], respectively. Fife et al. [20] additionally applied a self-controlled case series design and a modified case control design to the data set with extended confounder control and the removal of the requirement of an ophthalmologist visit resulting in no increased relative risk of FQ exposure.

Moreover, cohort studies from US comparing FQ to macrolides and beta-lactams [21], from UK comparing FQ to beta-lactam antibiotics [22], and from Denmark comparing to non-users [23], reported no differences in the relative risk of retinal detachment. Additionally, two nested case-control studies from the US and Korea [24, 25] reported no evidence for FQ-associated retinal detachment. Likewise, recent self-controlled case series from Hongkong [26], US claims comparing FQ to amoxicillin, azithromycin, and sulfamethoxazole-trimethoprim [27], and from UK comparing FQ to cephalosporins [28] reported no increased relative risk of FQ exposure.

Our cohort study with data from one of the largest German statutory health insurances contributed further real-world evidence against an increased relative risk of FQ exposure for retinal detachment based on a powerful number of retinal detachment cases and in comparison to all relevant reference antibiotics used in routine care. Moreover, we provided additional insights into patient characteristics and cannot depict any meaningful risk profiles for patients at high risk of retinal detachment occurrence post FQ exposure. Furthermore, we verified the absolute risk of retinal detachment in individuals with antibiotic prescriptions for Germany. Comparing all these studies, there may be a potential overestimation of FQ-associated retinal detachment in non-user comparisons. Moreover, the studies reporting an increased relative risk used data sets until 2010. Maybe in newer studies, such as in our analysis representing data from 2014 to 2019, prescribing physicians may already have incorporated earlier warnings regarding FQ-associated relative risk of retinal detachment in their choice of antibiotic drugs [29].

However, like any other observational study, the current analyses are subject to certain limitations. By the application of an appropriate study design, we strove to address these limitations. The active comparator new-user design was used to decrease the likelihood of confounding by indication; this was necessary because the German health claims data provide no direct linkage of antibiotic prescriptions to indications. Thus, by using an active comparator design, the baseline hazard of the reference group is more comparable to the exposure group, since it can be assumed that all antibiotics have been dispensed to patients with a bacterial infection. However, non-random treatment allocation is still present. We adjusted our analyses for relevant covariates and used a 1:1 propensity score matching process to reduce confounding by baseline conditions and treatment allocation as good as possible. Additionally, a sensitivity analysis comparing every single AC agent to FQ was conducted to address confounding by indication. Sensitivity analyses for 30-, 60-, and 92-days follow-up were conducted based on inpatient outcome detection to check whether 365 days of follow-up period introduced a bias towards the null. If retinal detachment occurred in short time windows after exposure, the entire year of follow-up might be too long, but shortened follow-up periods with 30, 60 and 92 days using inpatient diagnoses as outcome showed no differences, too. However, antibiotic prescription dispensing as exposure definition is just a proxy and does not ensure compliance with drug therapy. Non-adherence or missing compliance should be considered as potential factors of exposure misclassification. German statutory health insurance data do not cover covariates for lifestyle factors. Nevertheless, the analysed AOK data set is characterised by high completeness in terms of billing-relevant data itself and is informative for the assessment of the real health situation of patients at a population level in Germany with a meaningful number of covered individuals. This enables powerful effect estimations with high precision even for rare events such as retinal detachment. Lastly, the same study design, was used to successfully investigate FQ-associated outcomes several times [30] and to provide new insights into patient risk profiles in German data.

## Conclusion

In this cohort study, there was little evidence for FQ-associated retinal detachment. The absolute risk of retinal detachment after antibiotic exposure is generally low and there is no difference in the relative risks between different active comparator antibiotic agents and FQ episodes. No meaningful differences were observed for shortened risk windows as well as age and gender subgroups.

## Electronic supplementary material

Below is the link to the electronic supplementary material.

Supplementary Material 1

## Data Availability

The data that support the findings of this study are available from Scientific Institute of the AOK (WIdO) but restrictions apply to the availability of these data, which were used under license for the current study, and so are not publicly available. Statistical code available on request. The manuscript’s guarantors affirm that the manuscript is an honest, accurate, and transparent account of the study being reported; that no important aspects of the study have been omitted; and that any discrepancies from the study as planned have been explained.

## Abbreviations

AC: Active comparator
aHR: Adjusted hazard ratios
AOK: German statutory health insurance, called “Allgemeine Ortskrankenkasse”
ATC: Anatomic-therapeutic-chemical classification
CCI: Charlson comorbidity index
CED: Cohort entry date
DDD: Defined daily doses
EMA: European Medicines Agency
FQ: Fluoroquinolones
OPS: The German adaptation of the International Classification of Procedures in Medicine
PAMM: Piece-wise exponential additive mixed models
95% CI: 95% confidence intervals
