# Supplementary material for: Association between fluoroquinolones and retinal detachment: insights from a large German health claims-based cohort study
Source: BMC Ophthalmol. 2025 Aug 7;25:447. doi: 10.1186/s12886-025-04284-5 (PMC12329955; doi:10.1186/s12886-025-04284-5)
Supplement: Supplementary file 1 — Supplementary Material 1 [file 12886_2025_4284_MOESM1_ESM.docx]

**Supplement**

eTable 1. Definition of study variables

eTable 2. Study population characteristics after Propensity score matching

eTable 3. PAMM regression propensity score-matched cohort

eTable 4. PAMM subgroups and sensitivity analyses

eTable 5. PAMM active comparator agent-stratified analyses

eTable 1. Definition of study variables

| **Outcome** |  | **Codes** |
| --- | --- | --- |
| Retinal detachment: | ICD  OPS | H33.0, H33.2, H33.3, H33.4, H33.5 in combination with one of the following OPS codes:  5-152, 5-153, 5-154, 5-1560, 5-1561, 5-158, 5-159 |
| Covariates:  Diabetes mellitus  Cataract surgery  Endophthalmitis  Myopia  Other eye diseases  Serious eye trauma | ICD  OPS  ICD  ICD  ICD  ICD | E10, E11, E12, E13, E14  5-142, 5-143, 5-144, 5-145  H44.0, H44.1, H45.1  H44.2  H05, H30, H34, H35, H36, H43  S02.1, S02.3, S02.8, S05.1, S05.2, S05.3, S05.4,  S05.5, S05.6, S05.7, S05.8, S05.9, T26.2, T26.7 |

1 Anatomic-Therapeutic-Chemical (ATC) classification, the German adaptation of WHO-ATC classification, version 2020

2 International Classification of Diseases, 10th revision, German modification (ICD-10-GM), version 2020

3 “*Operationen- und Prozedurenschlüssel*” (OPS), the German adaptation of the International Classification of Procedures in Medicine, version 2020

eTable 2. Study population characteristics after Propensity score matching

|  | **After PS matching** | | |
| --- | --- | --- | --- |
|  | **FQ** | **AC** | **Standardised difference** |
|  | **(n= 2,925,881)** | **(n= 2,925,881)** |  |
| Age (mean (SD)) | 59.45 (19.47) | 59.45 (19.46) | <0.001 |
| Male gender (%) | 1,238,182 (42.32) | 1,234,774 (42.20) | 0.002 |
| CCI (%) |  |  | 0.003 |
| 0 | 1,471,844 (50.30) | 1,471,782 (50.30) |  |
| 1-2 | 832,472 (28.45) | 833,067 (28.47) |  |
| 3-4 | 380,748 (13.01) | 382,464 (13.07) |  |
| 5+ | 240,817 (8.23) | 238,568 (8.15) |  |
| Drugs dispensed (%) |  |  | 0.006 |
| 0 | 357,414 (12.22) | 355,799 (12.16) |  |
| 1-3 | 498,276 (17.03) | 495,207 (16.93) |  |
| 4-10 | 641,087 (21.91) | 638,776 (21.83) |  |
| 11-20 | 539,983 (18.46) | 539,053 (18.42) |  |
| 21+ | 889,121 (30.39) | 897,046 (30.66) |  |
| Hospitalised days (%) |  |  | 0.004 |
| 0 | 2,142,258 (73.22) | 2,146,914 (73.38) |  |
| 1-7 | 376,439 (12.87) | 375,241 (12.82) |  |
| 8+ | 407,184 (13.92) | 403,726 (13.80) |  |
| CED year (%) |  |  | 0.001 |
| 2014 | 656,302 (22.43) | 656,535 (22.44) |  |
| 2015 | 640,315 (21.88) | 640,600 (21.89) |  |
| 2016 | 604,220 (20.65) | 604,241 (20.65) |  |
| 2017 | 540,741 (18.48) | 541,001 (18.49) |  |
| 2018 | 484,303 (16.55) | 483,504 (16.53) |  |
| CED quarter (%) |  |  | 0.001 |
| Q1 (Jan.-Mar.) | 899,151 (30.73) | 900,343 (30.77) |  |
| Q2 (Apr.-Jun.) | 650,033 (22.22) | 649,834 (22.21) |  |
| Q3 (Jul.-Sep.) | 656,153 (22.43) | 654,979 (22.39) |  |
| Q4 (Oct.-Dec.) | 720,544 (24.63) | 720,725 (24.63) |  |
| Diabetes mellitus (%) | 688,165 (23.52) | 686,786 (23.47) | 0.001 |
| Cataract surgery (%) | 74,530 (2.55) | 65,835 (2.25) | 0.019 |
| Endophthalmitis (%) | 851 (0.03) | 412 (0.01) | 0.010 |
| Myopia (%) | 6,652 (0.23) | 3,755 (0.13) | 0.024 |
| Other eye diseases (%) | 389,492 (13.31) | 383,581 (13.11) | 0.006 |
| Serious eye trauma (%) | 8,200 (0.28) | 4,891 (0.17) | 0.024 |
| Propensity score (PS) \| standard deviation (SD) \| fluoroquinolone (FQ) \| active comparator (AC) \| Charlson Comorbidity Index (CCI) \| cohort entry date (CED) \| Quarter 1-4 (Q1-4): January-March, April-June, July-September, October-December.  None FQ episodes were dropped for matching. | | | |

eTable 3. PAMM regression propensity score-matched cohort

|  | | | **aHR** | **[95% CI]** | |
| --- | --- | --- | --- | --- | --- |
| FQ-episode (ref. AC) | | | 0.99 | [0.92;1.07] | |
| Males (ref. females) | | | 1.46 | [1.35;1.57] | |
| CCI (ref. 0) | | |  | | |
| 1-2 | | | 1.01 | [0.92;1.11] | |
| 3-4 | | | 1.01 | [0.89;1.14] | |
| 5+ | | | 0.88 | [0.75;1.04] | |
| Drugs dispensed (ref. 0) | | |  | | |
| 1-3 | | | 1.26 | [1.03;1.54] | |
| 4-10 | | | 1.29 | [1.07;1.56] | |
| 11-20 | | | 1.22 | [1.01;1.49] | |
| 21+ | | | 1.24 | [1.02;1.51] | |
| Hospitalised days (ref. 0) | | |  | | |
| 1-7 | | | 1.04 | [0.93;1.16] | |
| 8+ | | | 1.10 | [0.98;1.24] | |
| CED year (ref. 2014) | | |  | | |
| 2015 | | | 1.14 | [1.01;1.28] | |
| 2016 | | | 1.06 | [0.94;1.19] | |
| 2017 | | | 1.17 | [1.04;1.32] | |
| 2018 | | | 1.12 | [0.98;1.27] | |
| CED quarter (ref. Q1 (Jan.-Mar.) |  | | | |  |
| Q2 (Apr.-Jun.) | | | 0.99 | [0.89;1.10] | |
| Q3 (Jul.-Sep.) | | | 0.99 | [0.89;1.10] | |
| Q4 (Oct.-Dec.) | | | 1.02 | [0.92;1.13] | |
| Diabetes mellitus | | | 0.87 | [0.79;0.95] | |
| Cataract surgery | | | 2.12 | [1.83;2.45] | |
| Endophthalmitis | | | 13.25 | [7.87;22.32] | |
| Myopia | | | 2.16 | [1.38;3.39] | |
| Other eye diseases | | | 2.62 | [2.40;2.88] | |
| Serious eye trauma | | | 2.04 | [1.24;3.38] | |
|  | |  |  |  |  |
|  | | | edf | p-value | |
| Follow-up | | | 2.868 | <0.001 | |
| Age in years | | | 5.728 | <0.001 | |

Propensity score (PS) | adjusted hazard ratio (aHR) with corresponding 95% confidence interval [95%CI] | fluoroquinolone (FQ) | active comparator (AC) | Charlson Comorbidity Index (CCI) | cohort entry date (CED) | Quarter 1-4 (Q1-4): January-March, April-June, July-September, October-December | effective degrees of freedom (edf).

eTable 4. PAMM subgroups and sensitivity analyses

| **Analysis** | **aHR_FQ_** | **[95% CI]** |
| --- | --- | --- |
| Per-protocol censoring | 1.06 | [1.00;1.13] |
| Excluding hospitalisation at baseline | 1.03 | [0.96;1.11] |
| Hospitalisation during follow-up: |  |  |
| 0 days | 1.03 | [0.94;1.11] |
| 1-7 days | 0.90 | [0.81;1.01] |
| ≥ 8 days | 1.15 | [0.96;1.37] |
| Females |  |  |
| ≤ 39 years | 0.57 | [0.33;1.00] |
| 40-69 years | 1.02 | [0.90;1.14] |
| ≥ 70 years | 0.96 | [0.83;1.11] |
| Males |  |  |
| ≤ 39 years | 1.08 | [0.69;1.69] |
| 40-69 years | 1.09 | [0.97;1.21] |
| ≥ 70 years | 0.98 | [0.85;1.13] |
| Defined daily dose: |  |  |
| Low | 0.90 | [0.71;1.13] |
| Medium | 1.00 | [0.92;1.08] |
| High | 1.09 | [0.97;1.23] |
| Inpatient only |  |  |
| ≤ 30 days follow-up | 1.02 | [0.79;1.32] |
| ≤ 60 days follow-up | 0.97 | [0.81;1.15] |
| ≤ 92 days follow-up | 0.94 | [0.82;1.09] |
| ≤ 365 days follow-up | 1.03 | [0.96;1.10] |
| Diagnoses |  |  |
| Retinal detachment incl. retinal tear/ break (rhegmatogenous ablatio retinae) [ICD-10-GM: H33.0] | 0.97 | [0.88;1.07] |
| Retinal tear/break without retinal detachment [ICD-10-GM: H33.3] | 1.03 | [0.93;1.15] |
| Other forms of retinal detachment [H33.2, H33.4, H33.5] | 1.06 | [0.92;1.20] |

Adjusted hazard ratio for fluoroquinolone-episode (aHR) | 95% confidence interval (CI) [lower confidence level; upper confidence level].

eTable 5. PAMM active comparator agent-stratified analyses

|  | **aHR_FQ_** | **[95% CI]** |
| --- | --- | --- |
| ***365-day risk window**** |  |  |
| Amoxicillin | 0.99 | [0.91;1.08] |
| Amoxicillin-clavulanic acid | 0.97 | [0.87;1.09] |
| Azithromycin | 0.98 | [0.88;1.09] |
| Cephalexin | 0.89 | [0.52;1.53] |
| Cefuroxime | 1.02 | [0.94;1.11] |
| Clindamycin | 1.00 | [0.90;1.10] |
| Sulfamethoxazole-trimethoprim | 0.95 | [0.83;1.08] |
| Doxycycline | 1.04 | [0.94;1.16] |
| ***92-day risk window***** |  |  |
| Amoxicillin | 0.91 | [0.75;1.11] |
| Amoxicillin-clavulanic acid | 1.15 | [0.88;1.51] |
| Azithromycin | 0.85 | [0.67;1.09] |
| Cephalexin | 0.81 | [0.25;2.60] |
| Cefuroxime | 0.92 | [0.76;1.12] |
| Clindamycin | 0.78 | [0.63;0.97] |
| Sulfamethoxazole-trimethoprim | 0.91 | [0.68;1.22] |
| Doxycycline | 1.00 | [0.79;1.27] |

Adjusted hazard ratio for fluoroquinolone-episode (aHR) | 95% confidence interval (CI) [lower confidence level; upper confidence level]. | * quarterly-based censored, outpatient and inpatient outcomes | ** calendar week-based censored inpatient outcomes only
